# Sex difference in the relationship of the Atherogenic index of plasma with coronary artery lesions in diabetes: a cross-sectional study

**DOI:** 10.1186/s12944-022-01767-y

**Published:** 2023-01-21

**Authors:** Yi Hu, Xuan Wang, Chaodi Luo, Tingting Zheng, Gang Tian

**Affiliations:** Department of Cardiology, First Affiliated Hospital of Xi’an Jiaotong University, Xi’an, 710061 Shaanxi China

**Keywords:** Coronary artery lesions, Type 2 diabetes mellitus, Atherogenic index of plasma, Sex difference

## Abstract

**Background:**

Coronary artery disease (CAD) tends to progress more rapidly in the type 2 diabetes mellitus (T2DM) population and may be associated with dyslipidemia. This study explored the relationship of the atherogenic index of plasma (AIP) to coronary artery lesions in the T2DM population of different sexes.

**Methods:**

The research included 737 individuals who underwent coronary angiography from 2018 to 2019. The included clinical data and coronary angiographic findings were analyzed in the study.

**Results:**

Among the included male patients, those with coronary artery disease had a higher adjusted AIP (aAIP). In correlation analysis, the Gensini score was positively and linearly correlated with the aAIP in male T2DM patients. An aAIP cutoff value of 1.17 was determined from the receiver operating characteristic (ROC) curve of aAIP versus CAD risk in the study population. After dividing the aAIP into two groups by the cutoff value of aAIP, the group with the lower value was used as the control for logistic regression analysis. The results showed that the risk of CAD and multivessel lesions was higher when the aAIP was higher in men with T2DM, and this positive association was not affected by HbA1c, age, or the presence of glucose-lowering therapy. The ROC curve suggested that the aAIP can predict CAD risk in male T2DM patients. However, no relationship was found in the included female patients.

**Conclusion:**

In male T2DM patients, AIP may serve as a reliable marker for coronary artery lesions.

**Supplementary Information:**

The online version contains supplementary material available at 10.1186/s12944-022-01767-y.

## Introduction

Coronary artery disease (CAD) threatens the health of people worldwide [1, 2]. The incidence of CAD is increasing, and there are 11 million CAD patients in China [3]. It is estimated that one-third of CAD patients have type 2 diabetes mellitus (T2DM), and the CAD morbidity and mortality of T2DM patients are higher [4, 5]. Despite rapid medical and pharmaceutical development, the most effective and inexpensive way to reduce disease risk remains rational prevention [6]. However, the symptoms of CAD in patients with T2DM are often unremarkable, and early detection is more difficult. There is no doubt that coronary artery lesions directly affect the occurrence and development of CAD. Therefore, to better reduce the incidence and mortality of CAD in T2DM patients, it is necessary to determine a method that can judge the degree of coronary artery lesions.

For T2DM patients, dyslipidemia, mainly manifested by low high-density lipoprotein cholesterol (HDL-C) and elevated triglycerides (TG), is a major cause of increased cardiovascular risk [7]. The atherogenic index of plasma (AIP), calculated by log (TG/HDL-C), correlates with the degree of insulin resistance and abnormal lipid metabolism [8, 9]. Previous research found that AIP was independently associated with CAD, coronary artery lesions, coronary artery calcification, and arterial stiffness and had some predictive value [10–14]. A recent meta-analysis also showed a higher AIP value for CAD in adults [15].

However, it is unclear whether AIP can reflect coronary artery lesions in T2DM patients. Whether there are differences by sex has also not been confirmed. This research explored the association of AIP with coronary artery lesions in T2DM patients and attempted to provide new ideas to better predict the risk of CAD in patients with T2DM for early intervention and treatment.

## Methods

### Study population

The inclusion criteria for the subjects were as follows: (1) patients who were hospitalized for coronary angiography from January 2018 to April 2019 at the First Affiliated Hospital of Xi’an Jiaotong University and (2) patients with T2DM. The exclusion criteria were as follows: (1) patients with incomplete data; (2) patients who had undergone coronary intervention; (3) patients with stenosis < 50% in all major coronary arteries but with diffuse lesions; and (4) patients with severe hepatic or renal insufficiency, severe valvular disease, cardiomyopathy, rheumatic heart disease, and malignant tumors. A total of 737 patients (male = 412, female = 325) were finally included. The human research committee of the First Affiliated Hospital of Xi’an Jiaotong University approved the research protocol. The research complies with the Declaration of Helsinki.

### Data collection

Sex, smoking status, history of hypertension, antidiabetic and insulin use status, body mass index (BMI), age, systolic blood pressure (SBP), and diastolic blood pressure (DBP) were obtained from records. Serological parameters including albumin (ALB), HDL-C, aspartate aminotransferase (AST), low-density lipoprotein cholesterol (LDL-C), fibrinogen (FIB), D-dimer, fibrinogen degradation products apolipoprotein (FDP), glycosylated hemoglobin (HbA1c), total cholesterol (TC), TG, white blood cell (WBC), evaluation of glomerular filtration rate (eGFR), lymphocyte, monocyte, neutrophils, and hemoglobin (Hb) were measured using fasting venous blood samples. Coronary angiography data were obtained from angiographic operation records.

### Definitions

CAD was considered to be present if there was 50% or more stenosis in one of the major coronary arteries. Controls were non-CAD patients without diffuse lesions. A history of T2DM, HbA1c greater than or equal to 6.5%, or the in-hospital glucose tolerance test is in line with the 2-hour blood glucose greater than or equal to 11.1 mmol/L was considered T2DM [16]. A multivessel lesion was defined when two or more major coronary arteries had 50% or more stenosis. To make AIP always positive, the result of TG divided by HDL-C was scaled by a factor of 10, and the log-transformation was used to obtain the adjusted AIP (aAIP) [17]. The Gensini score was calculated separately by two authors (Yi Hu and Xuan Wang) according to standard methods for assessing the degree of coronary artery lesions [18, 19], and the calculation process was not affected by other study parameters.

### Statistical analyses

Statistical analyses were performed with SPSS 18.0. Continuous variables that followed a Gaussian distribution are presented as the mean (standard deviation) and were analyzed by t test. The skewed distributed continuous variable was presented as median (interquartile range) and analyzed through the Mann–Whitney U test. U The categorical variable was presented as frequency (percentage) and analyzed by chi-square test. The correlation of the aAIP (as a continuous variable) with the Gensini score was explored by the Spearman correlation coefficient and multiple regression analysis. The predictive significance of the aAIP for CAD risk was shown by the receiver operating characteristic (ROC) curve. The optimal cutoff value of aAIP was calculated by subtracting the sum of sensitivity and specificity by 1. The relationship between the aAIP (as a categorical variable) and CAD and multivessel lesions was explored by logistic regression analysis. The patients were divided into two groups (low aAIP and high aAIP) by the cutoff value of aAIP, with the lower group as the control. The results of the regression analysis are expressed as 95% confidence intervals (95% CIs) and odds ratios (ORs). Further stratified analysis was used to explore the interaction between the aAIP and confounding factors such as age (age<65 or age ≥ 65), HbA1c level (HbA1c<7.5 or HbA1c ≥ 7.5), and treatment as well as the interaction between the aAIP and sex in different subgroups. The outcome was statistically significant at a two-sided *P* < 0.05.

## Results

### Baseline characteristics of the clinical profile and biochemistry

After strict screening according to the criteria, 737 patients were finally included, consisting of 412 male patients and 325 female patients. There were 536 CAD patients, 320 males and 216 females. Compared with female patients, male patients had a higher smoking proportion, BMI, DBP, aAIP, AST, HbA1c, eGFR, Hb, WBC, monocytes, and neutrophils but lower age, SBP, LDL-C, HDL-C, TC, FIB, FDP, and D-dimer. Whether in all patients, male patients or female patients, HbA1c, HDL-C, FIB, and FDP were higher and Hb was lower in CAD patients than in non-CAD patients. TG, D-dimer, the number of current smokers, monocytes, neutrophils, and aAIP were higher and ALB and eGFR were lower in all patients and male patients with CAD. In female patients, SBP, BMI, and AST were higher in CAD patients, while the presence of CAD did not affect the aAIP value (Table 1 This table should appear after this paragraph).

**Table 1:** Clinical and biochemical characteristics

	Total(*n* = 737)			Male(*n* = 412)			Female(*n* = 325)			
	Control(*n* = 201)	CAD(*n* = 536)	*P*1	Control(*n* = 92)	CAD(*n* = 320)	*P*2	Control(*n* = 109)	CAD(*n* = 216)	*P*3	*P*4
Smoking,n%	52(25.9)	224(41.8)	<0.001	49(53.3)	223(69.7)	0.003	3(2.8)	1(0.5)	0.217	<0.001
Hypertension,n%	132(65.7)	376(70.1)	0.242	58(63.0)	209(65.3)	0.688	74(67.9)	167(77.3)	0.067	<0.001
Age, years	60(10)	62(10)	0.072	58(10)	60(10)	0.201	63(10)	66(9)	0.005	<0.001
BMI,kg/m^2^	25.9(4.0)	25.4(4.1)	0.282	26.0(3.6)	26.0(3.6)	0.442	25.7(4.4)	24.8(3.8)	0.002	<0.001
DBP,mmHg	79(11)	78(11)	0.349	81(13)	79(11)	0.158	77(9)	77(11)	0.682	0.004
SBP,mmHg	132(18)	135(19)	0.046	131(17)	133(18)	0.570	133(18)	140(19)	0.004	<0.001
LDL-C,mmol/L	2.11(0.76)	2.13(0.83)	0.764	2.00(0.73)	2.09(0.82)	0.290	2.21(0.78)	2.19(0.84)	0.821	0.031
TG,mmol/L	1.34(0.92)	1.53(0.96)	<0.001	1.12(0.80)	1.55(0.97)	<0.001	1.47(0.94)	1.49(1.02)	0.466	0.553
HDL-C,mmol/L	1.00(0.33)	0.87(0.25)	<0.001	0.93(0.29)	0.82(0.21)	<0.001	1.04(0.32)	0.98(0.32)	0.027	<0.001
TC,mmol/L	3.71(0.88)	3.71(0.97)	0.968	3.49(0.85)	3.60(0.97)	0.342	3.89(0.87)	3.86(0.97)	0.807	<0.001
aAIP	1.12(0.25)	1.26(0.25)	<0.001	1.09(0.24)	1.31(0.23)	<0.001	1.14(0.26)	1.18(0.27)	0.170	<0.001
AST,U/L	19(10)	20(10)	0.035	21(12)	20(9)	0.817	18(8)	20(10)	0.024	0.014
HbA1c,%	7.1(1.2)	7.5(1.5)	<0.001	7.1(1.4)	7.6(1.5)	0.005	7.0(1.1)	7.4(1.5)	0.007	0.049
ALB,g/L	40.8(4.2)	39.9(3.5)	0.004	41.3(4.6)	40.0(3.5)	0.012	40.4(3.7)	39.8(3.6)	0.170	0.191
D-dimer, mg/L	0.40(0.30)	0.48(0.30)	0.039	0.32(0.20)	0.40(0.30)	0.003	0.50(0.35)	0.50(0.30)	0.317	<0.001
FIB,g/L	2.96(0.92)	3.19(0.99)	<0.001	2.64(0.84)	3.13(1.01)	<0.001	3.17(0.80)	3.27(1.00)	0.004	<0.001
FDP,mg/L	1.10(0.60)	1.24(0.67)	0.001	1.00(0.69)	1.20(0.66)	0.001	1.20(0.78)	1.36(0.80)	0.012	<0.001
eGFR,ml/min/1.73m^2^	100.64(14.00)	98.35(13.75)	0.045	103.94(13.32)	100.36(13.78)	0.027	97.86(14.02)	95.38(13.18)	0.118	<0.001
Hb,g/L	140(15)	137(17)	0.034	150(12)	144(15)	<0.001	131(13)	127(13)	0.006	<0.001
WBC, 10^9^/L	6.24(2.2)	6.46(2.36)	0.059	6.26(2.13)	6.64(2.53)	0.036	6.24(2.30)	6.28(2.40)	0.956	0.003
Monocyte, 10^9^/L	0.33(0.14)	0.34(0.16)	0.001	0.34(0.13)	0.36(0.16)	0.002	0.32(0.15)	0.31(0.14)	0.417	<0.001
Lymphocyte, 10^9^/L	1.72(0.73)	1.61(0.71)	0.160	1.62(0.68)	1.60(0.70)	0.586	1.80(0.86)	1.63(0.73)	0.242	0.092
Neutrophils, 10^9^/L	3.97(1.74)	4.28(1.90)	0.011	3.96(1.68)	4.51(1.93)	0.013	3.99(1.79)	4.08(1.80)	0.633	<0.001
Antidiabetic,n%	106(52.7)	314(58.6)	0.153	44(47.8)	181(56.7)	0.138	62(56.9)	133(61.6)	0.415	0.142
Insulin,n%	37(18.4)	113(21.1)	0.422	23(25.0)	67(20.9)	0.406	14(12.8)	46(21.3)	0.064	0.257

P1 = reflect baseline characteristics between patients with and without CAD in all participants; P2 = reflect baseline characteristics between patients with and without CAD in males; P3 = reflect baseline characteristics between patients with and without CAD in females; P4 = reflect baseline characteristics between males and females

*CAD* coronary artery disease, *BMI* body mass index, *DBP* diastolic blood pressure, *SBP* systolic blood pressure, *aAIP* adjusted atherogenic index of plasma, *ALB* albumin, *HDL-C* high-density lipoprotein cholesterol, *LDL-C* low-density lipoprotein cholesterol, *FIB* fibrinogen, *FDP* fibrinogen degradation products apolipoprotein, *HbA1c* glycosylated hemoglobin, *AST* aspartate aminotransferase, *Hb* hemoglobin, *TG* triglyceride, *TC* total cholesterol, *WBC* white blood cell, *eGFR* evaluation of glomerular filtration rate

### Risk factors affecting Gensini score in CAD patients

In both male CAD patients and female CAD patients, the Gensini score was positively and linearly correlated with FIB (all *P* < 0.05). In male CAD patients, the Gensini score was positively and linearly correlated with LDL-C and aAIP (all P < 0.05). The Gensini score was weakly correlated with age and HbA1c in female CAD patients (all *P*<0.1), but the change in aAIP did not correlate with the Gensini score (both *P*>0.05) (Table 2 This table should appear after this paragraph).

**Table 2:** Spearman’s correlation and multiple regression analysis for the Gensini score in CAD patients

	Male (n = 320)				Female (n = 216)			
Parameters	R	*P*	B	*P*	R	*P*	B	*P*
Age	0.052	0.350	−0.233	0.544	0.153	0.025	0.749	0.071
HbA1c	0.060	0.282	1.483	0.453	0.116	0.089	3.273	0.083
LDL-C	0.246	<0.001	12.791	<0.001	−0.078	0.255	−4.295	0.191
FIB	0.215	<0.001	9.027	0.010	0.241	<0.001	11.052	0.002
aAIP	0.134	0.017	0.115	0.039	0.104	0.126	14.517	0.143

Adjusted for BMI, age, smoking, DBP, SBP, AST, ALB, FIB, D-dimer, FDP, HbA1c, eGFR, Hb, monocytes, and neutrophils

*CAD* coronary artery disease, *BMI* body mass index, *DBP* diastolic blood pressure, *SBP* systolic blood pressure, *aAIP* adjusted atherogenic index of plasma, *AST* aspartate aminotransferase, *LDL-C* low-density lipoprotein cholesterol, *ALB* albumin, *FIB* fibrinogen, *FDP* fibrinogen degradation products apolipoprotein, *HbA1c* glycosylated hemoglobin, *eGFR* evaluation of glomerular filtration rate, *Hb* hemoglobin

### Association of aAIP with CAD and multivessel lesions

**Fig. 1:**
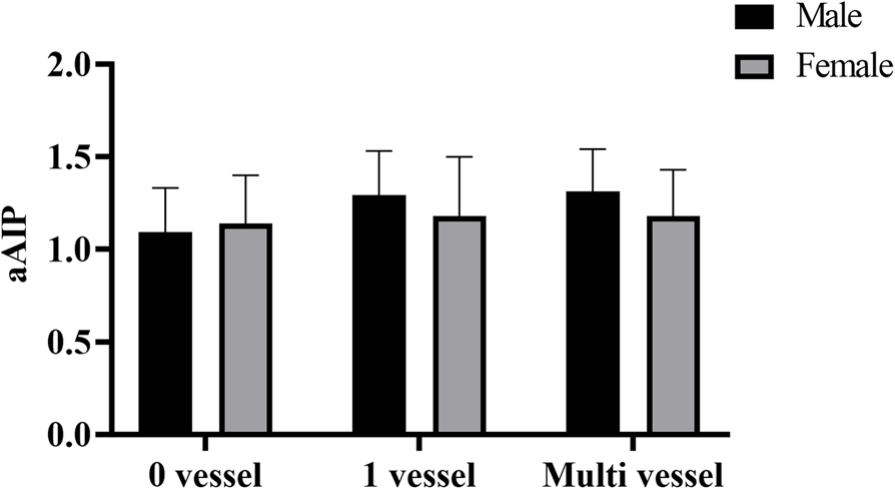
Association between aAIP and the number of coronary lesions. Analysis of variance showed significant differences in the aAIP between the 0-vessel lesion and the 1-vessel lesion and between the 0-vessel lesion and the multivessel lesion (all *P* < 0.05). Among the other groups, there was no difference in aAIP (all *P* > 0.05)

Analysis of variance showed the differences between the 0-vessel lesion group and the 1-vessel lesion, 0-vessel lesion, and multivessel lesion groups in male patients. There were no significant differences except for the above groups (Fig. 1). The area under the curve (AUC) revealed that the aAIP has predictive value for CAD (AUC = 0.651 *P* < 0.001) (Additional file 1: Fig. S1). According to the ROC curve, the optimal cutoff value for aAIP was 1.17. Therefore, aAIP < 1.17 was assigned to the low aAIP group, and aAIP ≥1.17 was assigned to the high aAIP group. Logistic regression analysis showed that patients with high aAIP had higher CAD risk and multivessel lesion risk. When no adjustment for confounders was performed, aAIP showed an OR of 2.800 for CAD (95% CI: 2.006-3.908, *P* < 0.001) and an OR of 2.054 for multivessel lesions (95% CI: 1.526-2.766, P < 0.001). After adjusting for confounders, aAIP showed an OR of 3.053 for CAD (95% CI: 2.068-4.506, *P* < 0.001) and an OR of 2.074 for multivessel lesions (95% CI: 1.465-2.935, *P* < 0.001) (Table 3 This table should appear after this paragraph).

**Table 3:** Logistic regression analysis of CAD and multivessel lesion risk

	Unadjusted		Adjusted	
	OR (95%CI)	*P*	OR (95%CI)	*P*
CAD risk	2.800(2.006-3.908)	<0.001	3.053(2.068-4.506)	<0.001
Multivessel lesion risk	2.054(1.526-2.766)	<0.001	2.074(1.465-2.935)	<0.001

Adjusted for BMI, age, sex, smoking, DBP, SBP, AST, LDL-C, ALB, FIB, D-dimer, FDP, HbA1c, eGFR, Hb, monocytes, and neutrophils

*CAD* coronary artery disease, *BMI* body mass index, *DBP* diastolic blood pressure, *SBP* systolic blood pressure, *AST* aspartate aminotransferase, *LDL-C* low-density lipoprotein cholesterol, *ALB* albumin, *FIB* fibrinogen, *FDP* fibrinogen degradation products apolipoprotein, *HbA1c* glycosylated hemoglobin, *eGFR* evaluation of glomerular filtration rate, *Hb* hemoglobin

### Association of aAIP with CAD and multivessel lesions in different subgroups

**Fig. 2:**
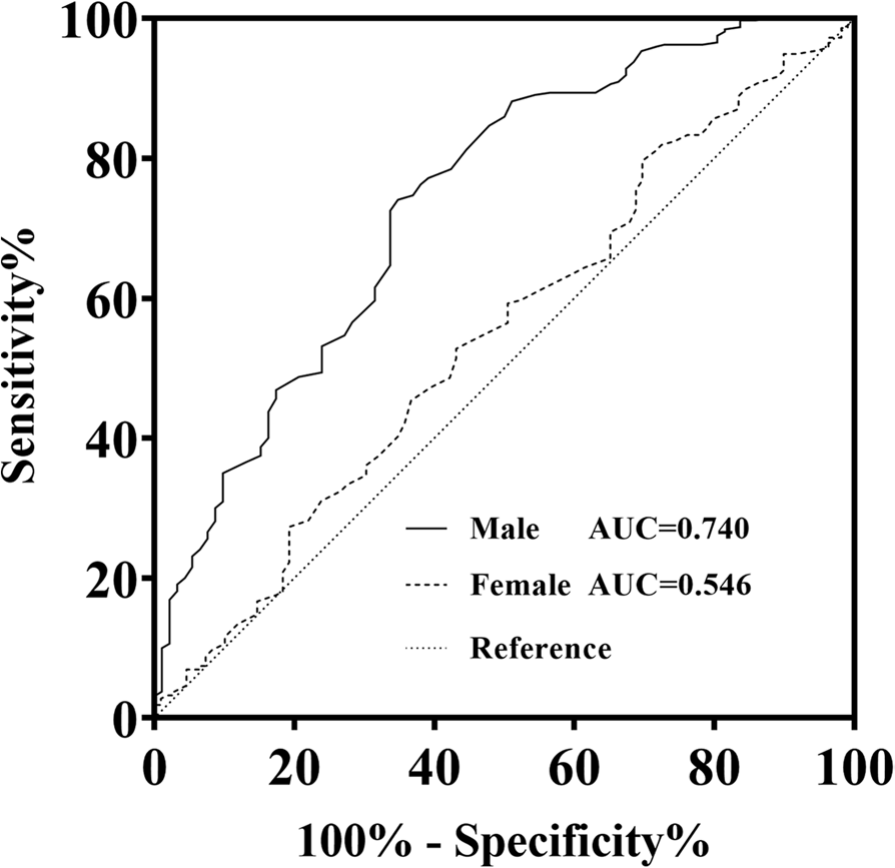
ROC curve analyses of the aAIP for predicting CAD risk in males and females

Multiple logistic regression showed that male patients with high aAIP had a higher risk of CAD (OR: 7.539, 95% CI: 4.032-14.097, *P* < 0.001) and multivessel lesions (OR: 2.951, 95% CI: 1.801-4.858, P < 0.001), but the association was absent in female patients (both *P* > 0.05). In addition, sex might interact with the association of the aAIP with CAD and multivessel lesions (both P < 0.001). Similar sex differences were obtained after stratification by age, HbA1c, and treatment status, and only in the elderly patient subgroup was no significant association between AIP and risk of multivessel lesions observed in either men or women. (Table 4 This table should appear after this paragraph). A multiple logistic regression analysis grouped by aAIP values was performed next. The results showed that among patients in the high aAIP group, males were more likely to have CAD (OR: 8.904, 95% CI: 3.463-22.898, *P* < 0.001) and multivessel lesions (OR: 5.454, 95% CI: 2.613-11.388, P < 0.001) than females. For patients in the low aAIP group, the risk of CAD was not significantly different between males and females, and the risk of multivessel lesions, although significantly different (OR: 2.254, 95% CI: 1.127-4.509, *P* = 0.022), was much smaller than that in the high aAIP group. However, no significant differences were found between the HbA1c, age, and treatment status, either in the low or high AIP group (all *P*>0.05) (Table 5 This table should appear after this paragraph). The AUC revealed that the aAIP has predictive value for CAD in male patients (AUC = 0.740 *P* < 0.001) but not in female patients (AUC = 0.546 *P*>0.05) (Fig. 2).

**Table 4:** Multiple logistic regression analysis of CAD and multivessel lesion risk in males and females

	N	CAD risk			Multivessel lesion risk		
		OR (95%CI)	*P*	*P* for interaction	OR (95%CI)	*P*	*P* for interaction
Total	737			<0.001			<0.001
Female	325	1.657(0.958-2.865)	0.071		1.550(0.920-2.613)	0.100	
Male	412	7.539(4.032-14.097)	<0.001		2.958(1.801-4.858)	<0.001	
HbA1c ≥ 7.5	300			<0.001			<0.001
Female	119	1.384(0.469-4.086)	0.556		1.334(0.510-3.492)	0.557	
Male	181	13.503(4.138-44.067)	<0.001		5.369(2.206-13.068)	<0.001	
HbA1c<7.5	437			<0.001			<0.001
Female	206	1.605(0.800-3.217)	0.183		1.683(0.859-3.295)	0.129	
Male	231	7.976(3.450-18.441)	<0.001		2.199(1.157-4.180)	0.016	
Age ≥ 65	303			<0.001			0.008
Female	171	1.455(0.638-3.317)	0.372		1.484(0.712-3.091)	0.292	
Male	132	9.277(1.715-50.173)	0.010		1.831(0.614-5.459)	0.278	
Age<65	434			<0.001			<0.001
Female	154	2.271(0.972-5.310)	0.058		2.097(0.903-4.870)	0.085	
Male	280	8.654(3.977-18.831)	<0.001		3.457(1.432-4.562)	<0.001	
Using antidiabetic/or insulin	486			<0.001			<0.001
Female	216	1.238(0.622-2.463)	0.543		1.299(0.684-2.467)	0.424	
Male	270	10.368(4.429-24.273)	<0.001		3.438(1.823-6.485)	<0.001	
Not using antidiabetic/or insulin	251			<0.001			<0.001
Female	109	1.587(0.505-4.989)	0.430		1.525(0.467-4.982)	0.485	
Male	142	11.856(3.082-45.614)	<0.001		2.883(1.086-7.659)	0.034	

Adjusted for BMI, age, smoking, DBP, SBP, AST, LDL-C, ALB, FIB, D-dimer, FDP, HbA1c, eGFR, Hb, monocytes, and neutrophils

*CAD* coronary artery disease, *BMI* body mass index, *DBP* diastolic blood pressure, *SBP* systolic blood pressure, *AST* aspartate aminotransferase, *LDL-C* low-density lipoprotein cholesterol, *ALB* albumin, *FIB* fibrinogen, *FDP* fibrinogen degradation products apolipoprotein, *HbA1c* glycosylated hemoglobin, *eGFR* evaluation of glomerular filtration rate, *Hb* hemoglobin

**Table 5:** ORs of patients with CAD and multivessel lesions in different AIP groups

	Low aAIP			High aAIP		
	N	OR (95%CI)	*P*	N	OR (95%CI)	*P*
CAD risk						
Sex	369			368		
Female	185	Reference		140	Reference	
Male	184	1.089(0.538-2.202)	0.813	228	8.904(3.463-22.898)	<0.001
HbA1c	369			368		
<7.5	246	Reference		191	Reference	
≥ 7.5	123	1.303(0.748-2.268)	0.349	177	1.361(0.741-2.500)	0.320
Age	369			368		
<65	192	Reference		242	Reference	
≥ 65	177	1.457(0.775-2.738)	0.243	126	0.639(0.304-1.345)	0.238
Antidiabetic/or insulin	369			368		
No	122	Reference		129	Reference	
Yes	247	1.214(0.691-2.131)	0.501	239	0.781(0.420-1.453)	0.436
Muti-vessel lesion risk						
Sex	369			368		
Female	185	Reference		140	Reference	
Male	184	2.254(1.127-4.509)	0.022	228	5.454(2.613-11.388)	<0.001
HbA1c	369			368		
<7.5	246	Reference		191	Reference	
≥ 7.5	123	1.237(0.725-2.112)	0.436	177	1.281(0.804-2.042)	0.298
Age	369			368		
<65	192	Reference		242	Reference	
≥ 65	177	1.750(0.952-3.215)	0.071	126	1.272(0.730-2.217)	0.395
Antidiabetic/or insulin	369			368		
No	122	Reference		129	Reference	
Yes	247	1.176(0.672-2.059)	0.569	239	1.075(0.664-1.741)	0.769

Adjusted for BMI, age, sex, smoking, DBP, SBP, AST, LDL-C, ALB, FIB, D-dimer, FDP, HbA1c, eGFR, Hb, monocytes, and neutrophils

*CAD* coronary artery disease, *BMI* body mass index, *DBP* diastolic blood pressure, *SBP* systolic blood pressure, *aAIP* adjusted atherogenic index of plasma, *AST* aspartate aminotransferase, *LDL-C* low-density lipoprotein cholesterol, *ALB* albumin, *FIB* fibrinogen, *FDP* fibrinogen degradation products apolipoprotein, *HbA1c* glycosylated hemoglobin, *eGFR* evaluation of glomerular filtration rate, *Hb* hemoglobin

## Discussion

Research results revealed a significant increase in Gensini score, risk of CAD and multivessel lesions with increasing AIP in male T2DM patients. However, in female T2DM patients, the study found that AIP was not associated with the Gensini score or the risk of CAD and multivessel lesions. The stratified analysis showed that the association of AIP with CAD and multivessel lesions was not influenced by confounding factors such as HbA1c level, age, and glucose-lowering treatment status. Nor was its sex difference influenced by these confounding factors. Therefore, in male patients with T2DM, AIP may be able to reflect the extent of coronary artery lesions, and monitoring AIP is expected to provide a noninvasive and convenient method for predicting coronary artery disease in men with T2DM.

In the study, women with CAD were older than men with CAD. Women are older than men at the time of CAD diagnosis due to differences in lifestyles, hormones, etc. [20–22]. DBP tends to be higher in men, SBP tends to be higher in women as they age, and hypertension is more closely associated with CAD in women than in men [23]. Furthermore, FIB, D-dimer and HDL-C were all higher in women with CAD than in men with CAD in the present study. This is consistent with previous studies showing that these indicators tend to be higher in women when cardiovascular disease is present [24–26]. Thus, the population characteristics in this study are similar to those in previous studies, which facilitates the generalization of our findings.

It is well known that dyslipidemia can promote atherosclerosis, in which LDL-C plays an important role [27]. The primary goal of most lipid-lowering drugs is also to lower LDL-C [28]. However, the cardiovascular risk remains considerable after LDL-C reduction [29]. Especially in patients with T2DM, LDL-C is often normal, so LDL-C may not be sufficient to predict CAD lesions in T2DM patients [9]. Numerous studies have revealed that hypertriglyceridemia, often accompanied by low HDL-C and high small dense LDL (sdLDL), can significantly contribute to atherosclerosis and may be an important contributor for CAD patients without high LDL-C [30, 31]. In addition, hypertriglyceridemia was highly correlated with several genes of CAD severity in T2DM patients [32]. According to the formula, a high AIP represents an elevation in TGs and/or a reduction in HDL-C in patients. There is growing evidence suggesting that AIP may indirectly represent sdLDL, a subclass of LDL that is increasingly recognized as a marker of CAD [33, 34]. Therefore, AIP has great potential as a predictive index for coronary artery lesions in T2DM patients.

The findings showed a strong association between AIP and the risk of multivessel lesions in male patients, whereas this association was not significant in older male patients. This may be related to the small sample size of elderly men or may be explained by the fact that longer-term disease progression in elderly patients with CAD is more likely to involve more coronary arteries, thus diluting the value of AIP. It is noteworthy that this study showed no association between AIP and coronary artery lesions in females with T2DM. First, the study did not discuss premenopausal versus postmenopausal women separately, which may have influenced the findings. A study with 4644 postmenopausal women found that AIP in postmenopausal women was associated with CAD risk [35]. However, there were also some studies with opposite results [17, 36]. Second, WBC, neutrophils, and monocytes were significantly higher in men than in women in this study, and some studies have proven that these indicators are closely related to CAD [37, 38]. Third, the protective effect of HDL-C may contribute to the sex difference. This protective effect is not only reflected in the numerical value of HDL-C but may also be related to the HDL-C function of the individuals [39]. Although male and female patients had similar AIP, they did not have consistent HDL-C values or function. Therefore, the specific mechanism needs to be further investigated.

### Comparisons with other studies and what does the current work add to the existing knowledge

The AIP can reflect cardiovascular risk in patients with T2DM, including prediction of CAD risk and prognosis of cardiovascular events [9, 14, 40]. In addition to analyzing CAD risk, the study further demonstrated that the degree of coronary artery lesions was related to AIP by assessing the Gensini score and the quantity of major coronary lesions, with sex differences. Thus, the present study further demonstrated that AIP could predict coronary severity in patients with T2DM, which is an important addition to previous studies and an important insight for future studies. Moreover, sex stratification is necessary when assessing coronary lesions in patients with T2DM by AIP.

### Strengths

On the one hand, the subjects of this study were patients with T2DM. Few studies have explored the association of AIP with coronary artery lesions in T2DM patients. It is also uncertain whether there are sex differences. On the other hand, multivariate analysis of multivessel lesion risk increased the study’s credibility.

### Limitations

The limitations of the study need to be recognized. First, the causal relationship between AIP and coronary artery lesions cannot be explained due to the limitations of the study methodology. Second, most of the patients were from Northwest China, and all of them were actively seeking medical care. Therefore, whether the findings are equally applicable to other regions, ethnicities, or nonattending patients requires more extensive studies. Finally, the calculation of AIP relies on laboratory findings of a single hospitalization. However, a single AIP may be confounded by lipid-lowering therapy and diet levels, and better designed prospective studies are needed to exclude confounding factors.

## Conclusions

In conclusion, AIP was significantly related to the degree of coronary artery lesions only in male patients with T2DM. For patients with T2DM with high AIP, especially for men, CAD prevention and screening should be carried out as soon as possible. Even if CAD is not diagnosed in these patients, a low-fat diet, regular exercise, etc., should be encouraged. In addition, future research on new lipid-lowering drugs may take sex into account.

## Supplementary Information

**Additional file 1.**

## Data Availability

The data involved can be viewed in the article.

## Abbreviations

aAIP: adjusted atherogenic index of plasma
CAD: coronary artery disease
T2DM: type 2 diabetes mellitus
BMI: body mass index
DBP: diastolic blood pressure
SBP: systolic blood pressure
LDL-C: low-density lipoprotein cholesterol
HDL-C: high-density lipoprotein cholesterol
ALB: albumin
HbA1c: glycosylated hemoglobin
FIB: fibrinogen
WBC: white blood cell
AST: aspartate aminotransferase
FDP: fibrinogen degradation products apolipoprotein
Hb: hemoglobin
TG: triglyceride
TC: total cholesterol
eGFR: evaluation of glomerular filtration rate
AUC: area under the curve
ROC: receiver operating characteristic
95% CI: 95% confidence interval
OR: odds ratio.
