# Supplementary figures and images for: Sex difference in the relationship of the Atherogenic index of plasma with coronary artery lesions in diabetes: a cross-sectional study
Source: Lipids Health Dis. 2023 Jan 21;22:10. doi: 10.1186/s12944-022-01767-y (PMC9862548; doi:10.1186/s12944-022-01767-y)

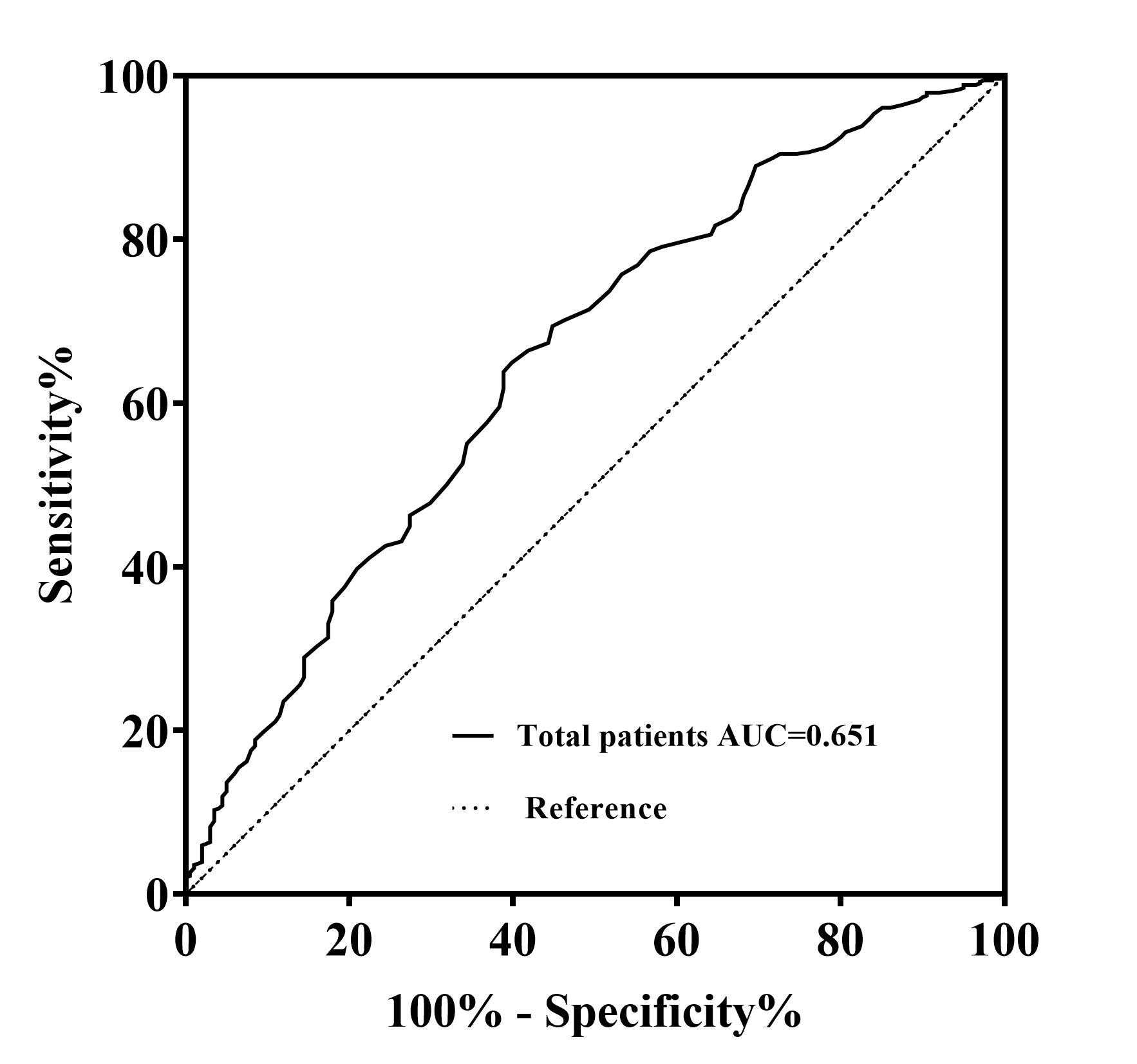

Supplement: Supplementary file 1 — Additional file 1. [file 12944_2022_1767_MOESM1_ESM.tif]
